# The role of neurophysiological tools in the evaluation of ischemic stroke evolution: a narrative review

**DOI:** 10.3389/fneur.2023.1178408

**Published:** 2023-04-27

**Authors:** Francesco Motolese, Jacopo Lanzone, Antonio Todisco, Mariagrazia Rossi, Francesca Santoro, Alessandro Cruciani, Fioravante Capone, Vincenzo Di Lazzaro, Fabio Pilato

**Affiliations:** ^1^Department of Medicine and Surgery, Unit of Neurology, Neurophysiology, Neurobiology and Psichiatry, Università Campus Bio-Medico di Roma, Rome, Italy; ^2^Fondazione Policlinico Universitario Campus Bio-Medico, Rome, Italy; ^3^Istituti Clinici Scientifici Maugeri IRCCS, Neurorehabilitation Unit of Milan Institute, Milan, Italy

**Keywords:** neurophysiology, TMS, EEG, collateral circulation, stroke, penumbra

## Abstract

Ischemic stroke is characterized by a complex cascade of events starting from vessel occlusion. The term “penumbra” denotes the area of severely hypo-perfused brain tissue surrounding the ischemic core that can be potentially recovered if blood flow is reestablished. From the neurophysiological perspective, there are local alterations—reflecting the loss of function of the core and the penumbra—and widespread changes in neural networks functioning, since structural and functional connectivity is disrupted. These dynamic changes are closely related to blood flow in the affected area. However, the pathological process of stroke does not end after the acute phase, but it determines a long-term cascade of events, including changes of cortical excitability, that are quite precocious and might precede clinical evolution. Neurophysiological tools—such as Transcranial Magnetic Stimulation (TMS) or Electroencephalography (EEG)—have enough time resolution to efficiently reflect the pathological changes occurring after stroke. Even if they do not have a role in acute stroke management, EEG and TMS might be helpful for monitoring ischemia evolution—also in the sub-acute and chronic stages. The present review aims to describe the changes occurring in the infarcted area after stroke from the neurophysiological perspective, starting from the acute to the chronic phase.

## Introduction

The first studies regarding cerebral perfusion in humans date back to the 1950s. Finnerty and coworkers demonstrated that when cerebral blood flow (CBF) drops below 29 mL/100 g/min, neurological impairment occurs (1). Some years later Jennet et al. showed that hemiparesis consistently occurred when relative cortical CBF was less than 30% compared with the baseline level (2) and subsequently, studies on vessel occlusion in animal models identified a critical threshold—i.e., 18 mL/100 g/min—for irreversible brain tissue damage (3). However, it is now clear that different individual factors contribute to tissue vulnerability after vessel occlusion, such as age, the brain structural reserve, inter-individual characteristics and collateral circulation. Indeed, adequate collateral blood flow limits the size of the infarct core in favor of the ischemic penumbra, i.e., the severely hypo-perfused and hypoxic brain tissue surrounding the core, that can be potentially saved if reperfusion occurs. In recent years there have been substantial advances in acute stroke management, regarding the extended time window for reperfusion therapies (4). The rationale is to select patients with large ischemic penumbra and small infarct using perfusion imaging.

However, perfusion imaging offers a “snapshot” in time of cerebral blood flow and it is not capable of capturing the evolution of stroke. MRI and CT scan provide several short-term prognostic parameters (e.g., hypoperfusion intensity ratio, Tmax, relative cerebral blood volume), reflecting the potential risk of infarction in the absence of reperfusion and, indirectly, the degree of collateral circulation (5, 6). The pathological process of stroke does not end after the acute phase, but it determines a long-term cascade of events, including changes of cortical excitability, that are quite precocious and might precede clinical evolution. These changes are difficult to detect with conventional neuroimaging, while electrophysiological techniques allow to capture the dynamic nature of stroke and thanks to their time resolution might be employed for repetitive evaluations. To this end, Electroencephalogram (EEG) or transcranial-magnetic stimulation (TMS) have been used as monitoring or prognostic tools in different stages of cerebrovascular disease.

EEG signal arises from synchronized synaptic activity in populations of cortical neurons. Studies on animal models have demonstrated that EEG actually reflects the cerebrovascular reactivity after vessel occlusion in the penumbra, while TMS might capture the reorganization of cortical circuits and the changes in functional connectivity due to plasticity mechanisms in later stages. Hence, electrophysiological techniques might be complementary to neuroimaging in the functional and structural evaluation of the brain after stroke.

In this review, we will investigate the potential employment of neurophysiological tools (e.g., EEG, TMS) to evaluate from a functional perspective how the changes in cerebrovascular reactivity influence the evolution of brain damage over time.

## The pathological evolution of brain infarction

**Figure 1:**
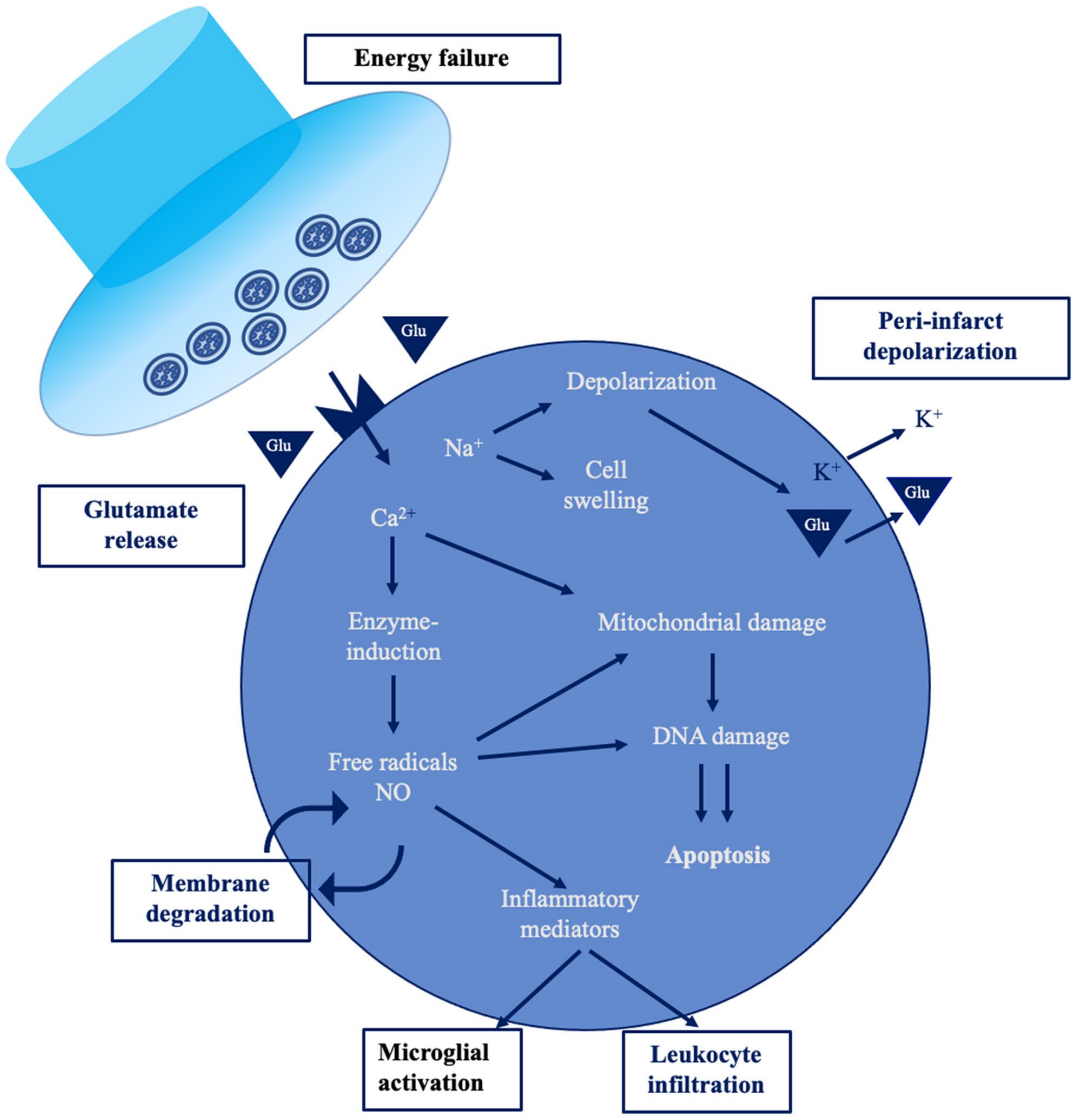
The ischemic cascade [modified from Endres et al. (7)]. This figure shows a simplified overview of the main pathological mechanisms occurring during acute ischemic stroke.

Cerebral ischemia occurs when blood flow to the brain is insufficient to meet metabolic demands. This might be focal—when a vessel supplying blood to the brain is obstructed—or global, e.g., in case of cardiac arrest. In stroke, the reduction of oxygen and glucose supply to energy-hungry brain cells—mainly neurons, but also glial cells—produces a cascade of time-dependent effects (Figure 1). The maintenance of ionic gradients and membrane potential of neurons consumes a great amount of energy, thus the reduction of CBF affects significantly brain oscillations. Neurons exhibit an individual vulnerability to hypoxia and the signal power of high frequency waves are first to be decreased at the early stage of stroke (8). If blood flow is interrupted for more than 20–60 s, synaptic dysfunction occurs, leading to neural function suppression (9). The absence of oxygen and glucose induces membrane ATPase failure and, consequently, a large Na + and Ca2+ intracellular influx with a massive neuronal depolarization. If oxygen shortage persists, the activation of calcium-dependent enzymes stimulates cell catabolism and induces cell death. In addition, glutamate release activates NMDA and AMPA receptors, exacerbating the intracellular calcium concentration, and producing excitotoxicity (10). Hypoxia damages the blood–brain barrier (BBB) and promotes the early migration of neutrophils (within 30 min) and lymphocytes (within 24 h), inducing inflammation (11, 12).

From the neurophysiological standpoint, the ischemic area is electrically silent, while the release of calcium and excitatory neurotransmitters generates a peri-infarct depolarization (PID) propagating across the surrounding area. This is not just a stroke epiphenomenon, but also induces calcium accumulation favoring delayed secondary pathology and neuronal death (13). Neurons in the penumbra are functionally impaired, so that they are “freezed”—i.e., electrically silent due to membrane potential imbalance—but anatomically preserved, as first described by Astrup and colleagues in the early ‘80s (14). Inflammation and apoptosis represent the main mechanism of damage within the ischemic penumbra in case of prolonged blood flow restriction, together with the production of detrimental oxygen-reactive species, ionic imbalance, protease activation, and DNA disruption (15).

In the acute phase, arterial collateral remodeling provides a compensatory supply since the sudden distal drop of pressure due to vessel occlusion generates an increase in flow within collateral circuits, namely “collateral recruitment” (16). If this mechanism is effective, the damage is limited.

In the subacute stage, the role of excitotoxicity and oxidative stress decreases while glial activation and neuroinflammation become more important (15). After 2 weeks, an immature glial scar begins to seal and compartmentalize the area of infarction from the surrounding parenchyma. About 7 weeks after, the process of gliosis culminates into a mature glial scar, defining the chronic phase of ischemic stroke (17).

For the scope of the present review, in agreement with the Stroke Roundtable Consortium, we designated the time after stroke as “acute” within the first 7 days, “subacute” within 6 months and “chronic” after 6 months (18). However, we have to acknowledge that a major limitation of literature regarding neurophysiology and stroke is the lack of a standard definition of stroke timepoints and the great heterogeneity of papers in this regard.

## Neurophysiological tools to monitor stroke evolution

Neural oscillations allow short- and long-term communication among neurons in the brain. At cortical level, they can be observed as large-scale oscillations in EEG signal. However, human networks have been studied *in-vivo* by other tools such as functional magnetic resonance imaging (fMRI) or noninvasive brain stimulation (NIBS) techniques (e.g., TMS) (19).

EEG is a non-invasive tool that has been frequently applied for stroke diagnosis and prognostication (20–22). EEG reflects extracellular currents resulting from excitatory and inhibitory post synaptic currents of cortical pyramidal cells. Quantitative EEG (qEEG) measures have been used as a more standardized approach to predict outcomes in ischemic stroke (23, 24), and qEEG metrics include the frequency spectrum analysis and topographic mapping. Other parameters can be derived from the EEG power spectrum. For instance, ratios of absolute power of different frequency bands, such as ratio of delta/alpha power, have shown to have a significant correlation with the clinical status—thus allowing a better categorization of stroke severity—and are also considered reliable prognostic values.

NIBS techniques explore functional alterations due to stroke on cortex excitability and on plasticity propensity (25). Besides, NIBS might be applied to evaluate connectivity. TMS is a non-invasive and painless technique which, when applied over the primary motor cortex (M1), generates a descending volley in the corticospinal pathway, and elicits a motor evoked potential (MEP) in the target muscles of the contralateral limb (26). Over the last 30 years, TMS has been widely used to study the underlying pathophysiology of various disorders, optimizing single-pulse, paired-pulse and repetitive stimulation protocols (27). TMS has been widely used in the acute phase after stroke to investigate changes in neural circuits and to provide information about cortical excitability, the cortical reorganization phenomena and to predict functional recovery.

A recent evolution of TMS technology allows to record the output of magnetic stimulation directly at the scalp using EEG. TMS-EEG has been used on stroke patients to probe cortical structural integrity and brain connectivity. Indeed, TMS-EEG elicits the so-called TMS evoked potentials (TEPs), characterized by positive and negative waveforms, that are indirect measures of functional integrity of cortical structures (28) (Table 1).

**Table 1:** Overview of the reviewed sources.

Stage	Authors	Neurophysiological evaluation
Acute	Finnigan et al. (32)	*EEG*
Acute	Shreve et al. (33)	*EEG*
Acute	Cohen et al. (34)	*EEG*
Acute	Ferreira et al. (35)	*EEG*
Acute	Phan et al. (36)	*EEG*
Acute	Agius et al. (37)	*EEG*
Acute	Cuspineda et al. (39)	*EEG*
Acute	Bentes et al. (40)	*EEG*
Acute	Jiang et al. (41)	*EEG*
Acute	Cuspineda et al. (42)	*EEG*
Acute	Aminov et al. (43)	*EEG*
Acute	Finnigan et al. (46)	*EEG*
Acute	Ajčević et al. (47)	*EEG*
Acute	Biskamp et al. (48)	*EEG*
Acute	Johnston et al. (49)	*EEG*
Acute	Schleiger et al. (50)	*EEG*
Acute	Ioroi et al. (51)	*EEG*
Acute	Pennisi et al. (52)	*TMS*
Acute	Rapisarda et al. (53)	*TMS*
Acute	Smith et al. (54)	*TMS*
Acute	Heald et al. (55)	*TMS*
Acute	Delvaux et al. (56)	*TMS*
Acute	Escudero et al. (57)	*TMS*
Acute	Stinear et al. (58)	*TMS*
Acute	Manganotti et al. (59)	*TMS*
Acute	Ahonen et al. (60)	*TMS*
Acute	Traversa et al. (61)	*TMS*
Acute	Classen et al. (62)	*TMS*
Acute	Cicinelli et al. (63)	*TMS*
Acute	Catano et al. (64)	*TMS*
Acute	Oozumi et al. (65)	*TMS*
Acute	McDonnell et al. (66)	*TMS*
Acute	Liepert et al. (67)	*TMS*
Acute	Swayne et al. (68)	*TMS*
Acute	Di Lazzaro et al. (69)	*TMS*
Acute	Tscherpel et al. (70)	*TMS-EEG*
Acute-Chronic	Gray et al. (71)	*TMS-EEG*
Acute	Russo et al. (72)	*TMS-EEG*
Subacute	Giaquinto et al. (73)	*EEG*
Subacute	Assenza et al. (74)	*EEG*
Subacute	Stinear et al. (75)	*EEG*
Subacute	Bütefisch et al. (76)	*TMS*
Subacute	Casula et al. (77)	*TMS-EEG*
Subacute	Finnigan et al. (78)	*EEG*
Chronic	Lanzone et al. (79)	*EEG*
Chronic	Vatinno et al. (80)	*EEG*
Chronic	Saes et al. (81)	*EEG*
Chronic	Cassidy et al. (82)	*EEG*
Chronic	Pirovano et al. (83)	*EEG*
Chronic	Graziadio et al. (84)	*EEG*
Chronic	Bembenek et al. (85)	*EEG*
Chronic	Stinear et al. (86)	*EEG;TMS*
Chronic	Madhavan et al. (87)	*TMS*
Chronic	Sivaramakrishnan et al. (88)	*TMS*
Chronic	Blicher et al. (89)	*TMS*
Chronic	Rolle et al. (90)	*TMS-EEG*
Chronic	Sarasso et al. (91)	*TMS-EEG*
Chronic	Hordacre et al. (92)	*TMS-EEG*

## Neurophysiological changes during the acute phase

The first hours after ischemic stroke are of paramount importance in terms of survival and long terms functional prognosis. Given the critical relevance of this phase, a lot of effort has been put to identify the best diagnostic and prognostic tools and to better understand the neuronal changes occurring in acute stroke. From the neurophysiological perspective, after vessel occlusion there are local changes—reflecting the loss of function in the infarcted area—but also widespread changes in neural networks, since structural and functional connectivity is disrupted (29). Changes in neural networks activity accurately reflect the blood flow in the affected area. These changes are highly dynamic and might indicate the improvement or the worsening of perfusion of brain tissue. Prompt restoring of blood flow in viable tissue stops the ischemic pathological cascade, improving local and global functional connectivity (30, 31).

### EEG studies

**Figure 2:**
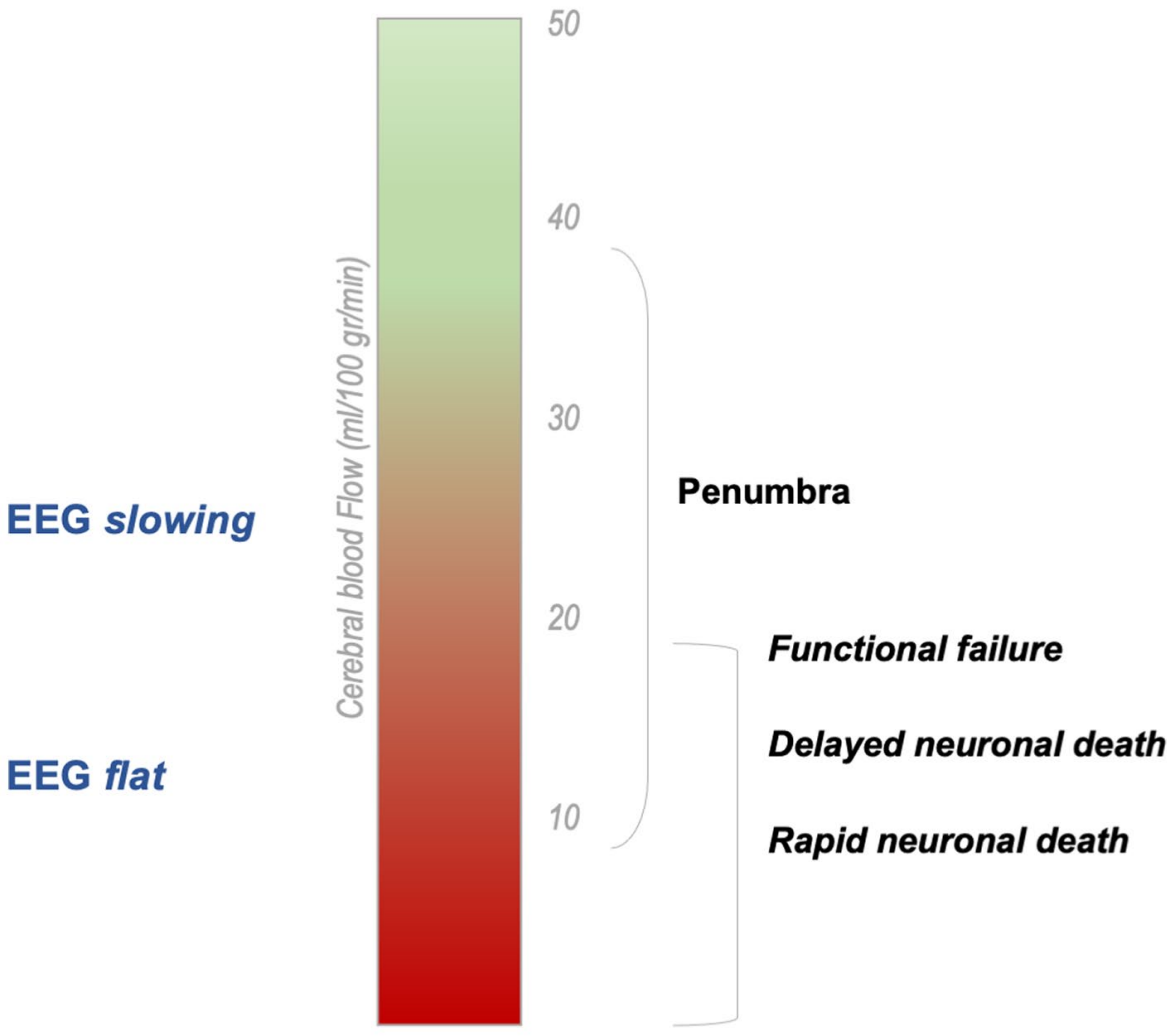
Cerebral blood flow related to Electroencephalographic findings in acute stroke [modified from Hofmeijer and Van Putten (9)]. In acute ischemic stroke, EEG signal changes according to CBF reduction. The infarcted area—where the damage is irreversible—is typically electrically silent on EEG.

Immediately after vessel occlusion, high-amplitude slow activity—in the delta frequency band (1–3 Hz)—appears in the involved brain regions (22, 32–34) (Figure 2). At intermediate level of ischemia—i.e., penumbra tissue—EEG changes might be less dramatic, including attenuation of beta activity and alpha slowing (21). In a single report of an animal model of ischemia, a significant increase of alpha band power during vessel occlusion has been found, immediately followed by the marked increase of delta power (35). Delta activity is the hallmark of cerebral dysfunction and has been reliably correlated with lesion location on neuroimaging (22). In fact, it is particularly evident on fronto-temporo-central electrodes after middle cerebral artery stroke (36).

Vascular insults produce an imbalance in the frequency activity between hemispheres since in the affected side there is a reduction of higher frequency activity and the increase of low-frequency bands. The brain asymmetry index (BSI) is a biomarker of motor functioning and recovery after stroke. Higher values of BSI—indicating a more pronounced asymmetry—are observed in acute stroke patients with a trend to normalization with spontaneous recovery (37). Most data indicate that the reestablishment of a balanced high frequency activity between motor areas predicts favorable motor recovery (28). However, the role of controlesional hemisphere is still debated and might depend upon stroke type and deficits (38).

A greater delta and theta activity within 24 h from onset, together with decreased faster activity and a greater interhemispheric asymmetry are associated with poor modified Rankin Scale (mRS) at discharge and worse prognosis (39–43). Two recent studies aimed to correlate changes of qEEG measures with long terms prognosis in acute stroke patients that underwent mechanical thrombectomy (44, 45). The results have shown that delta power 24 h after mechanical thrombectomy and the interhemispheric delta-alpha ratio are the best prognostic markers to define prognosis, even when compared with CT perfusion values (44).

Power measures—and especially delta activity—are associated with regional CBF, so that delta is negatively correlated with CBF while alpha is positively correlated (20). These measures might dynamically change, reflecting blood flow status, since reperfusion promptly induces the improvement of qEEG parameters. After blood flow restoration, there is a sudden increase of theta, alpha, and beta waves bandpower (35), while delta activity drops significantly but might persist for a while (46). Reduction of delta activity before symptoms improvement within 20 min after r-tPA administration has also been reported (46, 47). For these reasons, continuous EEG has been suggested as a monitoring tool during thrombolysis and thrombectomy.

qEEG measures include more complex indices such as delta/theta ratio (DTR), the delta/alpha ratio (DAR), and the (delta + theta)/(alpha + beta) ratio (DTABR). In an animal model of middle cerebral artery occlusion, the aperiodic spectral exponents—i.e., a putative marker of disrupted, inefficient neural communication—in peri-infarct area increased transiently and this correlated to a better recovery (48). Indeed, measures that asses the 1/f shape of the EEG spectrum have been proposed as a comprehensive way to assess the excitation/inhibition balance that is inherently expressed in EEG (49). All these parameters seem promising for monitoring stroke evolution, since their changes reflect accurately blood flow status.

Higher values of DTR, DAR and DTABR are observed during ischemia and rapidly decrease after reperfusion (32, 35). Schleiger and colleagues (50) acquired EEG continuously during thrombectomy, observing a significant reduction of DAR within several minutes of middle cerebral artery reperfusion. This change in EEG signal preceded the improvement of clinical symptoms. Authors concluded that DAR can effectively and immediately index salvage of the penumbra and might help clinicians predict the clinical outcomes in combination with the evidence of reperfusion revealed by imaging.

It has been also observed that in animal models of hypoxic brain one of the first feature to develop after vessel occlusion is a rapid reduction of EEG signal amplitude, that persist even after reperfusion occurs, probably due to a “safety mechanism” to reduce neuronal metabolism and protect cells (51). However, in the same study the reduced electrocortical brain activity after blood flow restoration was associated with lower oxygen utilization, thus suggesting the potential long-term development of brain damage (51). In this regard, neuroprotective treatments might be useful to protect tissue from delayed injury mechanism and preserve plasticity propensity in this stage (93).

To conclude, there are multiple evidence that indicate a strong relationship between EEG frequency/amplitude characteristics, CBF, and cerebral metabolism (23) in the acute phase.

### TMS studies

During the hyper-acute and acute phase of stroke, TMS over the affected hemisphere often fails to elicit motor evoked potentials (MEP) (52, 53) and the persistent absence of MEP after stroke is considered a marker of poor prognosis (54). In patients with intact cortico-spinal tract and preserved MEP, the motor thresholds are typically higher (53), MEP amplitudes are smaller and cortico-motor conduction time (CMCT) is delayed (55, 56) in comparison with those recorded from the unaffected hemisphere or from healthy individuals. These findings correlate with the long-term functional outcome and predict poor motor recovery (52, 53, 57, 58), although exceptions have been reported (56, 59). Another prognostic factor is cortical silent period (CSP), that is often prolonged after stroke (60–63) and it is correlated to the development of spasticity (64, 65).

In the first hours after stroke, the balance between cortical inhibition and excitation is altered. Cortical excitability of the affected hemisphere is usually reduced in both early and chronic phases of stroke (66). Thus, in most cases, the balance between hemisphere shifts toward excitation, while activity in the local inhibitory circuits is reduced. Short-interval intracortical inhibition (SICI) (59, 67) and long-interval intracortical inhibition (LICI) (68)—markers of GABA_A_ and GABA_B_ activity, respectively—of the affected hemisphere are reduced in the acute phase, whereas intracortical facilitation (ICF)—indicating Glutamatergic activity—remains normal (67, 68). Also, Short-Latency Afferent Inhibition (SAI), a paired pulse TMS protocol marker of central cholinergic activity, is suppressed in the acute phase of stroke and persistent suppression might indicate a better prognosis (69). These findings suggest that there is—in most cases—a “disinhibition” of the affected hemisphere, probably to favor plasticity and functional recovery.

Data regarding controlesional hemisphere are more controversial, since most evidence confirm that cortical excitability of the unaffected hemisphere does not change during stroke (66), and only SICI has been found to be suppressed (59, 67). Data also suggest that normalization of SICI in the unaffected hemisphere seems to be associated with good recovery (59, 68). Moreover, evidence regarding hemispheric imbalance are debated, since unaffected hemisphere might contribute to stroke recovery—i.e., vicariation model of functional recovery—or might have a detrimental effect on lesioned hemisphere—i.e., competition model. In this regard, another factor should be considered, the so-called structural reserve, a term indicating the non-lesioned residual neural networks, which is a critical element in determining if the effect of the contralateral hemisphere is beneficial or detrimental (38).

TMS-EEG is a more reliable measure of cortical reactivity compared with conventional TMS, since it is not influenced by distal components of the nervous system. Indeed, in patients with brainstem injuries MEPs could not be elicitable while TEPs are usually present (28). There are few interesting reports on the modifications of TEPs in acute stroke patients, because this technique requires long and careful recording sessions. Available data suggests that TEPs from lesioned hemisphere are less complex, more local and with a longer latency (70), and the absence of N100 or higher amplitude and delayed P30 are associated with poor functional recovery (59, 71). In a case report by Russo et al., the sleep-like slow-wave TMS-EEG response of acute stroke seems to partially recover with longitudinal follow up, along with clinical improvement (72).

Takin together, this evidence shows that local changes in connectivity and intracortical disinhibition of the affected hemisphere might be helpful for the recruitment of remaining motor output in the acute stage, and subsequently favor synaptic plasticity to promote recovery.

## Neurophysiological changes during the subacute phase

During the subacute phase of stroke, the ischemic lesion becomes better defined, while in the brain tissue surrounding ischemic area, hypoperfusion might variably persist (94). During acute and subacute phase of stroke, the neuroradiological findings might not correlate with the clinical impairment, because of the dynamic changes of the hypoperfused area and the extent of the surrounding edema. As time passes, other mechanisms, such as neuroplasticity or the brain structural reserve, might influence the clinical presentation. Indeed, since neurons are organized in networks, clinical manifestations do not depend just on the loss of function of the lesioned area, but on the global impairment of medium and large-scale circuits. Focal brain lesions may functionally impair remote regions, a phenomenon known as “diaschisis,” in which the excitability and metabolism of the remote regions, including the hemisphere contralateral to the stroke side, are reduced (95, 96).

Finally, neuroinflammatory mechanisms and vasogenic edema due to tight junction disruption might influence lesion consolidation and thus neurological impairment.

The quantification of EEG and TMS changes in this stage is of critical importance for defining the extent of brain damage after stroke.

### EEG studies

EEG and qEEG measures might be employed in this stage as prognostic tools. Slower frequency might persist on the electrodes overlying the lesioned area even after the acute stage and the magnitude of this activity depends on infarct volume (22). In one study about subacute stage, even after reperfusion, whole EEG power was lower than that of the control group, and also the DTR, DAR, and DTABR indices remained relatively high (35). This probably indicates the persistence of the ischemic stunning of the brain and the disruption of neural networks in stroke later stages.

In subacute middle cerebral artery stroke, the reduction of the asymmetry in high frequency activity between affected and unaffected hemispheres was associated with better motor performances over time (73). The presence of higher BSI value in the subacute phase is a strong indicator of poor prognosis, in particular if delta band power is present in the contralateral hemisphere (74), while more balanced high-frequency activity between hemispheres indicate a better functional prognosis.

Then, the persistence of slow activity and hemispheric asymmetry is a marker of greater damage post-stroke and of poor prognosis.

### TMS studies

In the subacute phase of stroke, the recruitment of spared neural networks became progressively more important in compensating the clinical impairment. Indeed, cerebral reorganization is critical for functional recovery.

Even in this phase, the absence of MEP from lesioned hemisphere—either in case of cortical or subcortical location—is related to poor functional prognosis (61). After infarction, the clinical recovery is associated with increased excitability, while the persistence of reduced cortical excitability in the affected hemisphere—as observed by increased resting MT or reduced SICI—is negative correlated with recovery (66). Stinear and Byblow proposed a simple diagnostic algorithm to predict functional recovery at 6 months on the basis of various measures collected during the first 7–10 days after stroke (75). In this algorithm the absence of MEP response, with significant clinical impairment, likely predicts poor functional prognosis.

A single study regarding subacute stroke patients reported the abnormal decreased of SICI of both hemispheres, with abnormal IHI from affected to unaffected hemisphere and preserved IHI the other way around (97). This finding was associated with excellent recovery of motor function and was interpreted as an adaptive process supporting recovery.

Following stroke, there is the expansion of the cortical map—i.e., the number of sites from where a MEP is elicited—indicating a progressive spatial recruitment of perilesional neurons. Indeed, this effect becomes more evident in patients undergoing rehabilitation, where training stimulate plasticity processes (76). Of note, the alteration of cortical excitability and inter-hemispheric communication might persist in cases of suboptimal recovery. Casula and colleagues reported a remarkable interhemispheric imbalance in stroke patients within 6 months from onset as evaluated by TMS-EEG. Also, they found that better recovery of hand strength was associated with a more stable interhemispheric balance (77).

These results indicate that remodeling of neural circuits underlying neuroplasticity and the reestablishment of interhemispheric balance are essential for recovery.

## Neurophysiological changes during the chronic phase

The natural evolution of vascular insult in the chronic phase is the formation of a glial scar. In this stage the reorganization of neural networks keeps on promoting functional recovery, even if this effect becomes weaker as time passes. However, this neural circuits remodeling might also have detrimental effects. For instance, the disruption of communication among neural networks might consolidate, constituting a putative mechanism of cognitive deficits after stroke (8).

In chronic stage, perfusion imaging shows the persistence of hypoperfusion in the area surrounding ischemic core (94). A study from Walenski et al. did not find any significant changes over time of tissue perfusion, even in patients that underwent successful rehabilitation (98). Also, hypoperfusion of areas close to the ischemic lesion was shown to correlate with the clinical status of aphasic patients (99). These data suggest that alterations of post-ischemic perfusion tend to persist in perilesional areas and cerebrovascular reactivity does not always seem to sensibly improve over time. Rather, it is the remodeling process that promote recovery.

### EEG studies

EEG is an helpful instrument for the longitudinal observation of stroke (28). The alterations in the slow band, usually found in the EEG of stroke patients, were consistently shown to improve from the sub-acute to the chronic phase in patients with good recovery (22, 78). Also changes in the 1/f properties of the EEG spectrum are sensitive to stroke’s evolution from sub-acute to chronic (79). These findings were also associated with various degrees of clinical correlation (79, 80).

As already said, patients with higher interhemispheric imbalance in the acute stage have usually a worse prognosis. The persistence of higher values of BSI in the subacute and chronic stage is still a biomarker of poor functional recovery, especially for what concerns the motor system (81, 82).

Finally, EEG connectivity appears to be locally impaired in chronic stroke, with significant modifications in connectivity from the subacute to the chronic stage (83). In the chronic phase, the reduction of the beta band (12.5–30.0 Hz) oscillatory activity in the motor cortex is an index of motor impairment (84).

### TMS studies

Studies regarding cortical excitability in chronic stroke confirm the findings discussed for acute and subacute stage. In particular, the presence of a motor-evoked potential within the first 2 weeks after stroke seems to have a strong predictive value on recovery (85) and the presence of MEP was shown to predict the residual potential for functional improvements, even several months after stroke (86). For these reasons, the presence of MEP in chronic stroke patients seems to be a promising marker to tailor the best rehabilitative approach (75). However, the presence of lower limb MEP did not seem to predict more specific features, such as walking speed (87, 88).

Some degree of disinhibition might persist even >6 months after stroke, without being modified by short-term training (89). Once again, this could be beneficial since it favors structural and functional remodeling but it might also indicate a persistent alteration of cortical circuitry.

TMS-EEG can increase the signal-to-noise ratio of EEG using magnetic perturbation. There are interesting reports on the modifications of TEPs in chronic stroke patients (70, 71, 90), despite the long recording sessions required. TMS-EEG modifications in peri-ischemic areas shows a peculiar modification of the evoked potentials, that compared with the contralateral hemisphere, loses fast complex oscillations and takes the shape of a bistable slow wave (91). In this regard, a higher amplitude of the P30 TEP component in chronic stroke patients has been reported (92). Generally speaking, data indicate that higher cortical reactivity and more complex evoked oscillatory activity in the lesioned hemisphere predicts better functional prognosis (28).

## Conclusion

Neurophysiological tools might still play a role in the evaluation of stroke, even in the “Imaging is brain” era (100). Ischemic stroke is a dynamic process, in which a cascade of events takes place after vessel occlusion. Neurophysiological tools are of great value for capturing these changes over time thanks to the excellent time resolution, allowing the longitudinal evaluation till the chronic phase. However, the methodological heterogeneity of the literature has limited the diffusion of techniques such as EEG or TMS in daily practice.

Neurophysiology and neuroimaging should be then considered as complementary tools exploring the same event from two different perspectives. This regards also the viability of penumbra brain tissue during stroke and, indirectly, the blood flow status. More studies are warranted for getting a better insight into stroke pathophysiology. This is of critical importance for developing tailored rehabilitation approach.

## Author contributions

FM, JL, and FP contributed to the conception. FM, JL, MR, AT, FS, and AC drafted the paper. FC, VL, and FP revised it critically for important intellectual content. All authors contributed to the article and approved the submitted version.

## Conflict of interest

The authors declare that the research was conducted in the absence of any commercial or financial relationships that could be construed as a potential conflict of interest.

The reviewer AM declared a past co-authorship with the author FP to the handling editor.

## Publisher’s note

All claims expressed in this article are solely those of the authors and do not necessarily represent those of their affiliated organizations, or those of the publisher, the editors and the reviewers. Any product that may be evaluated in this article, or claim that may be made by its manufacturer, is not guaranteed or endorsed by the publisher.
